# Multifactorial Programs for Healthy Older Adults to Reduce Falls and Improve Physical Performance: Systematic Review

**DOI:** 10.3390/ijerph182010842

**Published:** 2021-10-15

**Authors:** Vânia Loureiro, Margarida Gomes, Nuno Loureiro, Agustín Aibar-Almazán, Fidel Hita-Contreras

**Affiliations:** 1 Department of Arts, Humanities and Sports, School of Education, Polytechnic Institute of Beja, 7800-295 Beja, Portugal; margarida.gomes@ipbeja.pt (M.G.); nloureiro@ipbeja.pt (N.L.); 2 ISAMB Research Centre, Faculty of Medicine, University of Lisbon, 1649-028 Lisbon, Portugal; 3 Department of Health Sciences, Faculty of Health Sciences, University of Jaén, 23071 Jaén, Spain; aaibar@ujaen.es (A.A.-A.); fhita@ujaen.es (F.H.-C.)

**Keywords:** fall, exercise, multifactorial falls risk assessment, prevention, elderly

## Abstract

The aim of this systematic review of randomized controlled trials (RCTs) was to investigate the effects of multifactorial programs on the rate of falls and physical performance in ≥60 years old adults. A systematic literature search was conducted in four databases (PubMed, Scopus, Web of Science and Cochrane Library). A total of 518 articles were identified in the initial search, and six RCTs were finally included. Articles written in English, Portuguese and Spanish and published from January 2009 to May 2020 were included in this study. The methodological quality of the included studies was evaluated by the PEDro scale. A total of 518 studies were identified in the initial search, six RCTs were finally included, and three reached a level 1 of evidence. The findings of this systematic review of RCTs suggest that a physical exercise program, especially exercise group activities, combined with health education or with fall risk home assessment, were the most effective multifactorial program in reducing the rate of falls, although the results were not conclusive in all the studies included. Significant beneficial effects were observed in physical performance, particularly when assessed as gait, mobility and balance, regardless of the components of multifactorial program or exercise. This inconsistency in the results, particularly regarding the rate of falls, together with the variability among the multifactorial programs, suggest that any conclusion must be drawn with caution.

## 1. Introduction

Population ageing is a public health problem mainly due to decreased fertility rate and increased life expectancy [1]. The number of people aged ≥ 60 years is increasing and is projected to be 9.7 billion by 2050 [2], with 425 million older adults aged ≥ 80 years [3]. These facts suggest that, in order to promote health and active aging, European societies must improve their strength of health, long-term care and welfare systems [4].

It has been reported that falls are the fourth cause of injury-related mortality, with approximately 30% of community-dwelling adults > 65 years falling each year [5,6], and 5–10% of all falls have important consequences [7] such as fractures and fall-related injuries [8]. Besides, falls and fall-related fear of falling have been shown to be related to restriction of the activities of daily living, loss of autonomy, cognitive deterioration or depression [9].

Falls have a multifactorial etiology in older people [5] and the differences in the rate of falls between different geographical regions may be due to the frequency of particular intrinsic risk factors for falls [10]. Therefore, identifying fall risk factors and designing multifactorial interventions are key for the prevention of falls and fall-related injuries [11,12].

Differential effects for older adults should be adapted according to functional capacity when the assessment tools used for the elderly currently do not show high significant predictive validity [13,14]. Risk profiles, fall risk factors and fall prevention interventions need to increase their general predictive capacity as a component of the primary strategy oriented to promote healthy aging and active lifestyle [15,16].

Several physical performance measures, such as gait and mobility, balance or muscle strength, can determine a person’s ability to perform various movements of the upper and lower extremities that are required to perform basic activities of daily living [17]. Fall risk factors are frequently classified as either intrinsic or extrinsic (external to the individual), and physical performance-related parameters are key intrinsic fall risk factors [5]. These parameters deteriorate with aging, and their impairment has a great impact on public health, since they are not only well-known fall risk factors, but they may also lead to a loss of independence and disability and are major causes of morbidity and mortality in older adults [18].

Physical and socioeconomic environments are changing the daily living habits, reducing the demands of physical activity (PA) [19]. Evidence exists that supports the benefits of physical activity as a prevention strategy of multiple chronic diseases, health conditions and their associated risk factors among older adults [20,21,22]. It has been established that well-designed exercise programs as a single intervention (i.e., strength, balance, walking or flexibility training) can prevent falls and fall-related injuries in older adults [23], and in recent years, new types of exercise have been suggested to positively contribute to the prevention of falls [24,25]. However, given the multiple factors that may increase the risk of a fall, a multifactorial intervention would be the best fall prevention strategy.

Multifactorial intervention programs that include the evaluation and detection of fall risk factors have been proven to be effective in the prevention of falls and fall-related injuries [12,16]. These interventions require an individual evaluation to identify possible fall risk factors and combine two or more components [26]. Given the multifactorial etiology of falls, it is very important to design different specific strategies according to the different fall risk factors [27]. Questions about patients’ medical history, performance-based measures and self-report measures have been shown to have the greatest predictive value to evaluate the risk of falls [11].

The objective of this systematic review of randomized controlled trials (RCTs) was to provide an analysis of the published data regarding the effects of multifactorial programs based on individual assessment of fall risk factors on the rate of falls and physical performance in older adults aged ≤ 60 years.

## 2. Materials and Methods

This systematic review was conducted following the Preferred Reporting Items for Systematic Reviews and Meta-Analysis (PRISMA) statement [28] and other systematic reviews [14,29].

### 2.1. Information Sources and Search Strategy

A systematic bibliographic search was carried out in PubMed, Cochrane Plus, Web of Science and SCOPUS databases and was limited to the English, Portuguese and Spanish languages (V.L., M.G., A.A.-A., N.L. and F.H.-C.). The search strategy was carried out with a keyword search: (fall risk * OR risk factor *) AND (physical activity OR physical endurance OR physical condition OR level of physical activity OR physical performance) AND (multifactorial). A systematic process was employed to guarantee that all important articles were obtained. A supplementary manual search of studies was performed to identify potential RCTs not recorded by electronic database searches.

### 2.2. Inclusion and Exclusion Criteria

Inclusion criteria: RCTs that investigated the effect of multifactorial programs, according to fall risk components, on the rate of falls and physical performance in healthy community-dwelling adults aged ≥ 60 years at risk of fall, published from January 2009 to May 2020. Fall was defined as a sudden, unintentional change in position causing an individual to land at a lower level, on an object, the floor or the ground, other than as a consequence of sudden onset of paralysis, epileptic seizure or overwhelming external force [30]. Studies were excluded if: (i) did not study fall-related variables or were mentioned only as abstracts; (ii) were not RCTs; (iii) did not evaluate physical activity; (iv) did not determined fall risk with a validated tool; (v) did not use a control group.

### 2.3. Selection Process and Data Extraction

First, duplicate articles and those for which a summary was not available were discarded. Two authors (V.L. and M.G.) independently selected abstracts. Titles and abstracts were screened according to the eligibility criteria previously showed, and then, full texts were reviewed (V.L. and M.G.). Discrepancies were resolved by consensus with a third author (F.H.-C.). Data extracted included authors; year of publication; studied population (including groups, mean age and percentage of women); study design; outcomes; fall risk factors measures; measure time points, dropout and adverse effects; results (Table S1).

### 2.4. Outcomes

The primary outcomes were the rate of falls and physical performance. Secondary outcomes included fear of falling and physical activity level.

### 2.5. Quality of the Included Studies

Two independent authors (M.G. and A.A.-A.) performed the analysis of the studies finally included according to the PEDro scale. The instrument consists of 11 items that evaluate external and internal validity, as well as interpretability, although the criterion number 1 was not used to calculate the final score. The PEDro scale has shown to be a valid measure to assess the methodological quality of clinical trials [31]. A PEDro score ≥ 6 indicates a level 1 of evidence (6–8: good; 9–10: excellent) and ≤5 corresponds to a level 2 of evidence (4–5: fair; <4: poor) [32]. The methodological quality of the articles included was assessed with this scale. Disagreements between authors were first resolved by means of discussion and, then, by consultation with a third author (F.H.-C.).

## 3. Results

### 3.1. Inclusion of Studies

**Figure 1:**
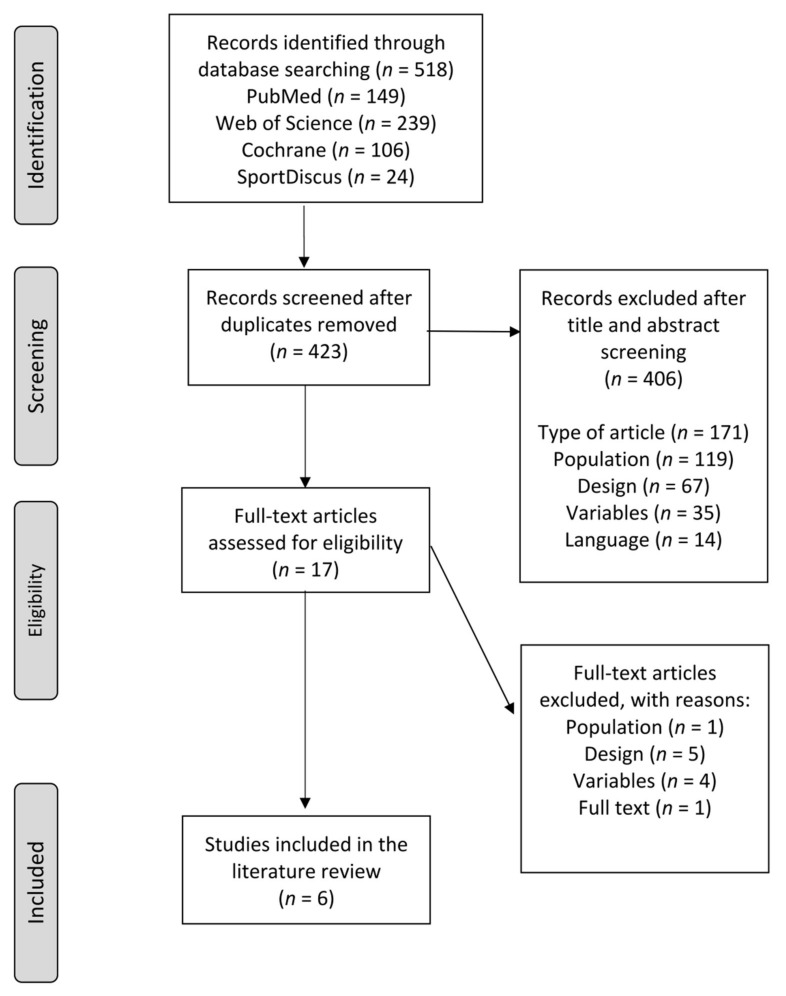
PRISMA flowchart showing the inclusion and exclusion in this systematic review.

The flow chart of study selection procedure, based on the PRISMA statement [28], is shown in Figure 1. Out of the 518 records identified in the initial search, a total of six articles were finally included in this systematic review [13,33,34,35,36,37].

### 3.2. Quality of Studies

The risk of bias assessment is shown in Table 1. A total of three articles [34,35,37] reached a level 1 of evidence (all PEDro scores were good), while the other three [13,33,36] obtained a level 2 (all PEDro scores were fair).

### 3.3. Characteristics of Studies and Participants

All the details and full descriptive findings of the studies that have been included in this review are presented in Table S1. This review included data from 2012 individuals, the sample size of the six studies included in this systematic review varied from 19 [33] to 616 [13] (54.4% female, 77.62 years). Four studies were conducted in Asia (Thailand [13] and Singapore [37]), two in Europe (Spain [33] and Germany [35]) and the other two in the United States of America [33] and Australia [35]. The contents of the multicomponent programs were strength and balance training (all studies), flexibility [13,33,34], endurance [13,37], gait [34,36] and functional [36] exercises and treatment of sensory impairments such as stimulation to use visual and somatosensory inputs [33].

### 3.4. Outcomes

#### 3.4.1. Rate of Falls

After the intervention periods, the incidence of falls was assessed at three months [33,34], six months [34], nine months [37] and twelve months [13,34,35,36]. The rates of falls (falls per participant) of the intervention groups were 0.05 (3 months), 0.06 (6 months), 0.31 (9 months) and 0.74 (12 months) falls per person and 0.10 (3 months), 0.11 (6 months), 0.38 (9 months) and 0.81 (12 months) falls per person in the control groups.

When analyzing the effects of the different components of multifactorial intervention (Table 2), the studies included in this review showed a variety of results. Significant improvements were observed when the intervention was limited to exercise with health education [36] and exercise with fall risk home assessment [33,34], although Pérula et al. [34] did not find significant results in the rate of falls (cumulative incidence) in the three follow-up visits. The studies that analyzed the multifactorial interventions with exercise programs, fall risk home assessment and medical management combined [35,37] did not show significant between-group differences, although Matchar et al. [37] reported that the risk of experiencing at least one injurious fall was statistically significantly lower in the intervention group. Lee et al. [13] did not find significant effects regarding fall rates with the combination of all interventions.

As for the way of delivering the exercise programs (Table 3), only group activities revealed a significant decrease in fall rates in comparison with the control group [33,36]. Home-based intervention [35] did not reveal significant results, and with the combination of home-based and group activities [13,34,37], only Pérula et al. [34] showed a significant reduction in the rate of falls.

#### 3.4.2. Physical Performance

Physical performance was assessed by mobility and gait [13,33,34,35,36,37], balance [13,33,34,35,36], strength [13,33,35,36] and physiological functioning [13,35]. Mobility was evaluated with the timed up and go test [13,33,36], the short physical performance battery [35,37] and the 4-min walk test [35]. Gait was analyzed by the GAIT Rite^®^ system [33] and the gait component of the performance-oriented mobility assessment (POMA) [34]. The analysis of the effects of the different multifactorial interventions according to their components (Table 2) revealed that exercise combined with health education [36], as well as fall risk home assessment associated with exercise programs and medical management, revealed statistically significant positive effects on mobility [35,37]. On the other hand, when the intervention combined an exercise program and fall risk home assessment [33,34], only Pérula et al. [34] demonstrated statistically significant improvements in gait. Lastly, Lee et al. [13], who studied the effects of all these four components of multifactorial intervention combined, described significant beneficial effects on mobility. Regarding the way of delivery of the exercise interventions (Table 3), mobility was improved after group activities [36] and home-based program [35], and the sum of both home-based program and group activities led to significant positive results in mobility [13,37] and gait [34].

Balance was assessed by the postural sway component of the physiological profile assessment (PPA) [13,35], the balance component of the POMA [34] and the modified Romberg test [37], as well as the Berg balance scale and the sensory organization test [33]. Strength was determined by the strength component of the PPA [13,35], manual muscle testing [33] and the chair–stand test [36].

The five studies that analyzed balance [13,35,36] found significant benefits regardless of the multifactorial intervention or the method of delivery of the exercise program (Table 2 and Table 3). Regarding strength, there were significant benefits in all the different combinations of multifactorial interventions [13,33,35], except when only exercise and health education were combined [36]. As for the mode of delivery of the exercise intervention, group activities [33], home-based program [35] and their combination [13] led to significant increases in strength. The PPA total score was used as a fall risk index [13,35], and significant benefits were reported only when the four components of the multifactorial intervention and the two ways of delivering the exercises were used together [13].

#### 3.4.3. Secondary Outcomes

The fear of falling and the PA level were analyzed by four [13,34,36,37] and two [13,34] of the studies included in this systematic review, respectively. A significant reduction in the fear of falling was reported in the participants that were involved combined exercises either in group activities alone (exercise and health education) [36] or together with home-based programs (exercise and fall risk home assessment) [34]. Lee at al. [13] observed that the fear of falling decreased in both groups after the intervention, but no overall group x time significant differences could be determined. With respect to PA level, Pérula et al. [34] described an increase in the weekly time spent on physical activity (66.2% of the participants of the intervention group), while Lee et al. [13] reported that the participants who were at a marked risk of falling and performed a combination of the four components of multifactorial intervention increased their PA level.

## 4. Discussion

The purpose of this systematic review was to summarize RCTs that evaluated the effects of multifactorial interventions based on individual assessment of fall risk factors on the rate of falls and physical performance in older adults aged 60 years and over. The results displayed four different combinations of multifactorial interventions: exercise intervention, fall risk home assessment, health education and medical management. The combination of a physical exercise program, particularly exercise group activities, and health education or fall risk home assessment appeared to be the most effective multifactorial program. On the other hand, beneficial effects on physical performance, mostly in gait, mobility and balance, were found after the interventions irrespective of the components of the multifactorial program or the method of delivering the exercise.

Multifactorial interventions are considered as a primary treatment strategy for fall prevention [38]. In a recent systematic review and meta-analysis, Hopewell et al. [39] concluded that these interventions, which usually include exercise, may reduce the rate of falls and suggest that this effect may be smaller when compared with usual care together with non-tailored falls prevention advice. Tricco et al. [40] reported that exercise alone and various combinations of interventions were associated with lower risk of injurious falls compared with usual care. The results of the present systematic review showed that a physical exercise program, combined with health education [36], or with fall risk home assessment [33,34], were the most effective in reducing the rate of falls.

When analyzing the way of delivery of the exercises, group activities [33,36] displayed significant results, but improvements were observed in home-based programs only when combined with group activities [34]. As for the rest of the RCTs, Fairhall et al. [35] found no effect on the rate of falling, but the authors explained that the sample size of their study was not powered for falls. On the other hand, Lee et al. [13] did not observe between-group difference in the rate of falls. Nevertheless, the participants in the control group had significantly fewer falls in the previous 12 months, and the fall incidence significantly decreased in the intervention group. Besides, Matchar et al. [37] reported that the rate of falls of the intervention group was lower but did not reach statistical significance after the intervention period; however, the risk of experiencing ≥1 injurious fall was significantly inferior in the intervention group.

Mobility alterations and gait problems can produce a series of physical, cognitive and social consequences for the elderly such as reduced independence or disability [41]. The results of the studies included in the present systematic review indicate that five out of six interventions that include physical exercise programs combined with another type of intervention reported significant improvements in mobility or gait. Only Beling and Roller [33] failed to obtain statistically significant improvements, although post-intervention timed up and go test mean score for the intervention group improved to below cutoff described by Shumway-Cook et al. [42] to identify older adults at risk for falls. Older adults with impaired balance are more prone to fall, and balance training is of great importance in fall prevention [43]. Among the findings of this systematic review, the studies that measured balance showed significant improvements after the intervention period. This may be due to the fact that strength exercises were introduced in all the intervention programs, and increased strength has been found to be associated with improved balance in older adults [44].

Muscle strength is very important in the performance of many activities of daily living. Reduced muscle strength is considered as an important predictor of the functional ability of the elderly [45], playing a key role in the diagnosis of sarcopenia and frailty, and it is related to a higher risk of falls [46]. In the present systematic review, three out of the four studies that analyzed strength found significant benefits [13,33,34]. Siegrist et al. [36] reported better chair–stand test scores after the intervention, but did not reach statistical significance, although baseline scores were significantly worse in the control group.

The PPA consists of five measures of physical functioning that assess muscle strength, balance, vision, reaction time and lower limb proprioception, and it has been shown to be predictive of falls [47]. Significant positive effects in the PPA total score were observed when the multifactorial program included the four different components, with both home-based and group activities [13]. On the other hand, Fairhall et al. [35] did not find an overall treatment effect in the PPA fall risk score after 12 months, but significant improvements were reported in strength and postural sway tests, which may be logical, since the participants performed a tailored home program of balance and lower limb strength training.

The fear of falling is a very important fall risk factor in older adults, but it may also lead to activity self-restriction, limitation of the activities of daily living and loss of autonomy in this population [48]. Different physical exercise interventions have been shown to have beneficial effects on the fear of falling [49,50]. The results of this systematic review are in agreement with these observations, and a significant reduction in the fear of falling was reported after multifactorial interventions that combined exercises either alone (exercise and health education) [36] or together with home-based programs (exercise and fall risk home assessment) [34]. Finally, physical activity is very important in older adult populations, and it has been showed that higher levels of total physical activity, at any intensity, are linked to substantially reduced risk for premature mortality [51]. Pérula et al. [34] described that 66.2% of the participants of the intervention group showed an increase in weekly physical activity. On the other hand, Lee et al. [13] reported that the participants of the intervention group with marked fall risk reported better PPA scores both within and between groups after the intervention period. However, there were no differences with respect to low and moderate fall risk levels, and as authors state, this similar level of physical activity might have partially accounted for the lack of difference regarding fall incidence.

This systematic review has some limitations. Although multifactorial programs are heterogeneous by nature, this variability should be taken into account when interpreting the results. The studies included also showed heterogeneity regarding the assessment time points (short, medium and long term). Besides, only three studies showed a good or excellent methodological quality, and the sample size of some RCTs may have restricted the possibility of finding significant results, especially regarding the rate of falls. In addition, only two out of the six studies included have provided information about adverse outcomes.

## 5. Conclusions

The results of this systematic review of RCTs that analyzed the effects of multifactorial interventions based on fall risk factors suggest that exercise training programs, particularly those performed in a group format, combined with other components such as fall risk home assessment or health education, are the most effective in reducing the rate of falls, although the results are heterogeneous. Improvements in physical performance were described in all the studies included, mostly in gait, mobility and balance, regardless of the type of components or the way of delivery of the exercises. The fact that only half of the studies included showed a level 1 of evidence and the quality of the studies included suggest, together with the heterogeneity among the types of multifactorial programs and exercises modalities, and the small number of articles finally included suggest that these results must be interpreted with caution. Future RCTs with a higher methodological quality level, an adequate sample size and more information regarding adverse effects are recommended.

## Figures and Tables

**Table 1:** List of included studies with PEDro scores.

Studies	(a)	(b)	(c)	(d)	(e)	(f)	(g)	(h)	(i)	(j)	(k)	Score
Beling and Roller [33]	Yes	Yes	No	Yes	No	No	No	No	No	Yes	Yes	4/10
Pérula et al. [34]	Yes	Yes	No	No	No	No	Yes	Yes	Yes	Yes	Yes	6/10
Lee et al. [13]	Yes	Yes	No	Yes	No	No	No	Yes	No	Yes	No	5/10
Fairhall et al. [35]	Yes	Yes	Yes	Yes	No	No	Yes	Yes	Yes	Yes	Yes	8/10
Siegrist et al. [36]	Yes	Yes	No	Yes	No	No	No	No	No	Yes	Yes	4/10
Matchar et al. [37]	Yes	Yes	No	Yes	No	No	Yes	Yes	Yes	Yes	Yes	7/10

(a) Eligibility criteria; (b) random allocation; (c) concealed allocation; (d) baseline comparability; (e) blinding of subjects (f) blinding of therapists; (g) blinding of assessors; (h) adequate follow-up; (i) intention-to-treat analysis; (j) between-group comparisons; (k) point estimates and variability.

**Table 2:** Effects of the different types of multifactorial intervention according to their components (*n* = 6).

		Components					
		Exercise + FRHA		Exercise + HE	Exercise + FRHA + MM		Exercise + FRHA + HE + MM
		Beling and Roller [33]	Pérula et al. [34]	Siegrist et al. [36]	Fairhall et al. [35]	Matchar et al. [37]	Lee et al. [13]
Rate of falls		✓ *	✓ *	✓ *	✓	✓	✓
Physical performance	Mobility and gait	✓	✓ *	✓ *	✓ *	✓ *	✓ *
	Balance	✓ *	✓ *	✓ *	✓ *		✓ *
	Strength	✓ *		✓	✓ *		✓ *
	Physiological functioning				✓		✓ *
Fear of falling			✓ *	✓ *			✓
PA level			✓				✓ *

* = *p* < 0.05|FRHA = fall risk home assessment; HE = health education; MM = medical management; PA = physical activity.

**Table 3:** Effects of different ways of delivery of the exercise interventions (*n* = 6).

		Ways of Delivery of Exercise Intervention					
		Group Activities		Home-Based Program	Group Activities + Home-Based Program		
		Beling and Roller [33]	Siegrist et al. [36]	Fairhall et al. [35]	Pérula et al. [34]	Matchar et al. [37]	Lee et al. [13]
Rate of falls		✓ *	✓ *	✓	✓ *	✓	✓
Physical performance	Mobility and gait	✓	✓ *	✓ *	✓ *	✓ *	✓ *
	Balance	✓ *	✓ *	✓ *	✓ *		✓ *
	Strength	✓ *	✓	✓ *			✓ *
	Physiological functioning			✓			✓ *
Fear of falling			✓ *		✓ *		✓
PA level					✓		✓ *

* = *p* < 0.05|PA = physical activity.

## Supplementary Materials

The following are available online at https://www.mdpi.com/article/10.3390/ijerph182010842/s1, Table S1: Characteristics of the included studies.
