# Supplementary material for: Multifactorial Programs for Healthy Older Adults to Reduce Falls and Improve Physical Performance: Systematic Review
Source: Int J Environ Res Public Health. 2021 Oct 15;18(20):10842. doi: 10.3390/ijerph182010842 (PMC8535839; doi:10.3390/ijerph182010842)
Supplement: Supplementary file 1 [file ijerph-18-10842-s001.zip › ijerph-1363981-supplementary.pdf]

Table S1. Characteristics of the included studies.

Study, year and location	Studied population, groups, Age (mean), women (%)	Study design	Outcomes	Fall risk factors measures	Measure time points, dropout and adverse effects	Results
Beling and Roller (2009) [33] United States of America.	n= 19.<br>IG= 11 (79.0±6.5 years, 36.4% women).<br>CG=8 (81.0±5.0 years, 50% women).	Exercise Intervention:<br>- Group activities: 12 weeks; 3 sessions/week; 1h/session; components: center of gravity exercises; balance strategies; stimulation of visual and somatosensory inputs; range of motion and strength exercises).<br>Falls risk home assessment.<br>CG Instructions: No intervention; after intervention CG was invited to a 12-week balance intervention.	Primary outcomes:<br>- Rate of falls.<br>- Mobility and gait (TUG test, GAIT Rite® system).<br>- Balance (BBS and SOT).<br>- Strength (MMT)	Cognition (cut off ≥24/30- MMSE)<br>Functional/Balance (cut off ≥13.5 seconds- Timed Up and Go test)<br>History of falls (2 or more falls in the previous year).	Measurements:<br>- At baseline.<br>- At 3 Months.<br>Dropout:<br>IG (n=1)<br>CG (n=3)<br>Adverse effects:<br>Not mentioned	Rate of falls: significantly lower in the IC compared with CG (p≤0.05).<br>TUG test, GAIT Rite® system: no significant differences.<br>BBS and SOT: significant between-group differences in BBS (p<0.05), and significant within-group results in SOT (p<0.05).<br>MMT: significant between-group differences in knee extension (p≤0.05) and significant within-group improvement in the IG regarding dorsiflexion (p≤0.05).
Pérula et al. (2012) [34] Spain.	n=404<br>IG=133 (76.35±3.9years, 59.4% women)<br>CG=271 (76.5±4.6years, 50.2% women)	Exercise Intervention:<br>- Group Activities: 1 health education session; physical exercise workshop: flexibility and muscle strength, balance, and gait; 5 sessions; 90 min/session; 3 weeks.<br>- Home-based program: exercises (at least 30 min, 4d/week: flexibility; muscle strength; balance and gait) and walking (at least 30 min/day; 7 times/week).<br>Falls risk home assessment.<br>CG Instructions: brief individual advice; information leaflet about fall prevention and usual clinical care.	Primary outcome:<br>- Rate of falls (FC).<br>- Mobility and gait (POMA-gait).<br>- Balance (POMA-balance).<br>Secondary outcomes:<br>- Fear of falling.<br>- Physical activity level (IPAQ).	WHO questionnaire (risk factors not available).	Measurements:<br>- At baseline.<br>- At 12 Months<br>Falls recorded at 3, 6. 12 months.<br>Dropout:<br>IG (n=12)<br>CG (n=13)<br>Adverse effects:<br>Not mentioned	Rate of falls: when compared to CG, the incidence of falls at home was significantly lower in the IG at 12 months (p=0.040). No significant results in the rate of falls (cumulative incidence) in the 3 follow-up visits.<br>POMA-gait: between-group significant differences (p=0.001).<br>POMA-balance: between-group significant differences (p=0.002).<br>Fear of falling: significant differences (p=0.002).<br>IPAQ: at 12 months (66.2% of the IG increased their PA)
Lee et al. (2013) [13] Thailand.	n=616<br>IG=313 (75.40±7.2 years, 53.0% women)<br>CG=303 (76.0±7.0 years, 56.1% women)	Exercise Intervention:<br>- Group Activities (supervised training, small groups; 8 weeks; 1 session/ week; 50-60 min/session; components: strength; balance; cardiorespiratory endurance; flexibility).<br>- Home-based program (Frequency: diary; 50-60 min/session; components: strength; balance; cardio-respiratory endurance; flexibility).<br>Falls risk home assessment: Home hazards: evaluation/modification.<br>Health Education: 4 sessions; education brochures.<br>Medical Management: ophthalmologist or other specialist. Medication review.<br>CG Instructions: health education brochures; medication review; medical referral.	Primary outcome:<br>- Rate of falls (PC, DP).<br>- Mobility and gait (TUG test).<br>- Balance (PPA-postural sway).<br>- Strength (PPA-strength).<br>- Physiological functioning (PPA-total score).<br>Secondary outcomes:<br>- Fear of falling (FES-I).<br>- Physical activity level (IPAQ).	PPA (vision, strength, peripheral sensation,) vestibular function.<br>Participants were classified in low, moderate and marked fall risk levels according to the PPA.	Measurements:<br>- At baseline.<br>- At 12 Months<br>Dropout:<br>IG (n=34)<br>CG (n=41)<br>Adverse effects:<br>not mentioned	Rate of falls: no differences were observed.<br>TUG test: IG showed significant differences (p<0.001), and compared to CG had a greater improvements (p<0.001).<br>PPA-postural sway: improvements in both IG and CG with open eyes (<0.001 and p=0.002 respectively) and close eyes (p=0.007 and p<0.001 respectively). With open eyes, IG had greater significant improvements than CG (p=0.026).<br>PPA-strength: both IG and CG showed significant improvements (p=0.008 and p=0.001, respectively). CG had greater significant improvements (p=0.003).<br>PPA-overall index: IG and CG showed significant improvements (p<0.001). Compared to CG, IG had significant improvemet (p=0.004).<br>FES-I and IPAQ: IG had only significant within-group improvements (p<0.001 and p=0.002,respectively).<br>According to PPA fall risk level: IG participants with marked risk had within and between-group improvements in PPA-postural sway with open eyes (p<.0001, p=0.006), TUG (p<0.001,p=0.001) and IPAQ (p<0.01, p=0.040).

Fairhall et al. (2013) [35] Australia.	n=241<br>IG=120 (83.4±5.8 years, 67.0% women)<br>CG=121 (83.2±5.9 years, 68.0% women)	Exercise Intervention:<br>- Home-based program (20–30 min/ session; 3 to 5 sessions /week; components: balance; lower limb strength).<br>Falls risk home assessment: home modifications.<br>Medical Management: medication review; management of chronic health conditions.<br>CG: usual care, medical management services and health involvement.	Primary outcomes:<br>- Rate of falls (FC, PC).<br>- Mobility and gait (SPPB and 4-m walk test),<br>- Balance (PPA-postural sway).<br>- Strength (PPA-strength).<br>- Physiological functioning (PPA-total score).	**Cardiovascular Health Study:**<br>Weight loss;<br>Fatigue;<br>Decreased grip Strength;<br>Slow gait speed;<br>Low physical activity.	Measurements:<br>- At baseline<br>- At 3 Months<br>- At 12 Months<br>Dropout:<br>IG (n=13)<br>CG (n=6)<br>Two intervention group participants experienced back pain consistent	At 3 months: no significant between-group differences were described.<br>At 12 months:<br>Rate of falls: no differences were observed when comparing IG and CG.<br>SPPB: compared to CG, IG showed significant better results ($p<0.001$).<br>4-m walk test: Participants of the IG walked significantly faster ($p=0.02$).<br>PPA-postural sway: the results of the IG were significantly better results those of the CG ($p=0.02$).<br>PPA-strength: IG had significantly better performance than CG ($p=0.03$).<br>PPA-total score: no significant differences.
Siegrist et al. (2016) [36] Germany	n=378<br>IG=222 (78.0 ± 6.0 years, 77.48% women)<br>CG=156 (78 ± 6 .0 years, 72.44% women)	Exercise Intervention:<br>- Group Activities (supervised training; 16 weeks; 1 session /week; 60 min/ session; components: strength; balance; functional training; gait.<br>Health Education: workshop: fall prevention; duration: 3.5 hours.<br>CG: no structured treatment to prevent falls.	Primary outcomes:<br>- Rate of falls (FC, PC).<br>- Mobility and gait (TUG).<br>- Balance (mRomberg).<br>- Strength (CST).<br>Secondary outcomes:<br>- Fear of falling (FES-I).	History of falls (1 or more falls in the previous year).<br>Physical function-TUG test.<br>Strength- CST.<br>Balance-mRomberg.<br>Fear of falling-FES-I.	Measurements:<br>- At baseline<br>- At 12 Months<br>Dropout:<br>IG (n=38)<br>CG (n=40)<br>No adverse effects were observed	Rate of falls: significant lower incidence ($p=0.007$) in the IG.<br>TUG: significant better results in the IG ($p=0.014$).<br>mRomberg: increased significantly in the IG($p=0.037$).<br>CST: no significant differences.<br>FES-I: reduced significantly in the IG ($p=0.022$).
Matchar et al. (2017) [37] Singapore	n= 354<br>IG=177 (78.2±6.9 years, 75.14% women)<br>CG=177 (74.4±7.2 years, 75.14% women)	**1º Active Intervention -3 months**<br>Exercise Intervention:<br>- Group Activities: exercise program -SAFE; 24 sessions; 1 hour/ session; 2 sessions/week; components: endurance; mobility; balance; integrative mobility training; lower leg strength.<br>- Home-based program:12 sessions; 3 times / week; specifically targets and modifies impairments.<br>Falls risk home assessment: Environmental Hazards - CDC home checklist.<br>Medical management: vision (self-report; Snellen chart and pinhole ocular occlude), polypharmacy (>4).<br>**2º Maintenance Phase**<br>CG: Usual care prescribed by a physician and educational materials on falls prevention.	Primary outcomes:<br>- Rate of falls (FC, PC)/ injurious falls.<br>- Mobility and gait (SPPB).	SPPB.	Measurements:<br>- At baseline<br>- At 9 Months<br>Dropout:<br>IG (n=15)<br>CG (n=16)<br>Adverse effects: not mentioned	Rate of falls: no statistically significant between-group differences at 9 months, but the risk of having at least 1 injurious fall was significantly lower in IG ($p=0.041$).<br>SPPB: The IG showed significant better results ($p=0.029$).

BBS= Berg Balance Scale; CDC= Centers for Disease Control and Prevention; CST=Chair-Stand-Test; CG= Control Group; DP= Department visits; FC= Falls Calendar; FES-I= Falls Efficacy Scale-International; IG= Intervention Group; IPAQ= International Physical Activity Questionnaire; MCT= Motor Control Test; MMT= Manual muscle testing; MMSE= Folstein mini-mental state examination; mRomberg: modified Romberg test; PC= Phone Call; POMA= Performance Oriented Mobility Assessment; PPA= Physiological Profile Assessment; ROM= Range of Motion; SAFE= Steps to Avoid Falls in Elderly; SOT= Sensory Organization Test; SPPB= Short Physical Performance Battery; TUG= Timed Up & Go Test; WHO= World Health Organization.
